# Supplementary figures and images for: Genome-Wide Identification and Expression Analysis of the Dof Transcription Factor in Annual Alfalfa Medicago polymorpha
Source: Plants (Basel). 2023 Apr 29;12(9):1831. doi: 10.3390/plants12091831 (PMC10181442; doi:10.3390/plants12091831)

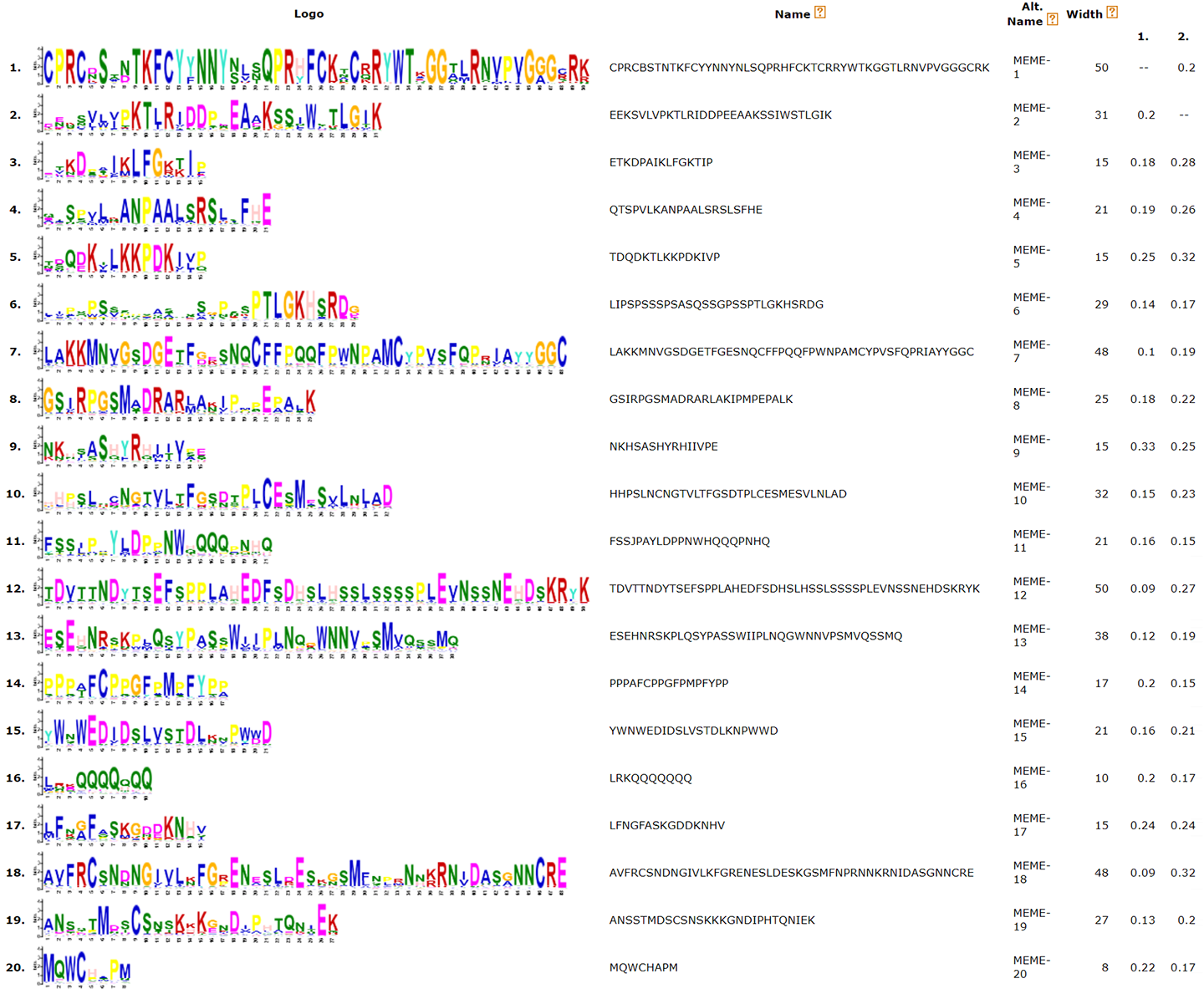

Supplement: Supplementary file 1 [file plants-12-01831-s001.zip › FigS1.tif]
